# Dimethyl 2,2′-[2,2′-bi(1*H*-1,3-benzimidazole)-1,1′-di­yl]diacetate

**DOI:** 10.1107/S1600536812032266

**Published:** 2012-07-21

**Authors:** Huai-Ling Guo, Jia-Cheng Liu, Chao-Hu Xiao, Ting Pang, Ping Cao

**Affiliations:** a Key Laboratory of Polymer Materials of Gansu Province, Key Laboratory of Bioelectrochemistry & Environmental Analysis of Gansu College of Chemistry and Chemical Engineering, Northwest Normal University, Lanzhou 730070, People’s Republic of China

## Abstract

The whole mol­ecule of the title compound, C_20_H_18_N_4_O_4_, is generated by an inversion center. The benzimidazole ring mean plane make a dihedral angle of 89.4 (8)° with the plane passing through the acetate group (COO). In the crystal, mol­ecules are linked *via* weak C—H⋯O hydrogen bonds and π–π inter­actions [centroid–centroid distance = 3.743 (3) Å] involving inversion-related benzimidazole groups.

## Related literature

For related structures, see: Al-Mohammed *et al.* (2012 ▶); Fu & Xu (2009 ▶); Xu & Wang (2008 ▶). For the synthesis of 2,2′-bibenzimidazole, see: Tang *et al.* (2007 ▶).
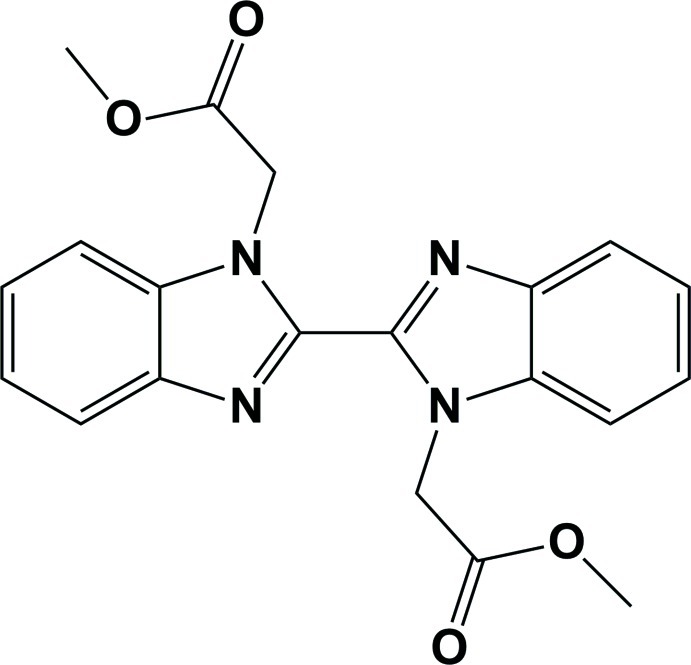


## Experimental

### 

#### Crystal data

C_20_H_18_N_4_O_4_
*M* *_r_* = 378.38
Triclinic, 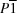

*a* = 6.904 (4) Å
*b* = 8.494 (5) Å
*c* = 8.643 (5) Å
α = 67.191 (5)°
β = 70.360 (5)°
γ = 87.172 (5)°
*V* = 438.1 (4) Å^3^
*Z* = 1
Mo *K*α radiation
μ = 0.10 mm^−1^
*T* = 296 K
0.33 × 0.31 × 0.29 mm

#### Data collection

Bruker APEXII CCD diffractometer
Absorption correction: multi-scan (*SADABS*; Bruker, 2005 ▶) *T*_min_ = 0.967, *T*_max_ = 0.971
3034 measured reflections
1600 independent reflections
1172 reflections with *I* > 2σ(*I*)
*R*_int_ = 0.027

#### Refinement

*R*[*F* ^2^ > 2σ(*F* ^2^)] = 0.048
*wR*(*F* ^2^) = 0.127
*S* = 1.06
1600 reflections
128 parameters
H-atom parameters constrained
Δρ_max_ = 0.14 e Å^−3^
Δρ_min_ = −0.25 e Å^−3^

### 

Data collection: *APEX2* (Bruker, 2005 ▶); cell refinement: *SAINT* (Bruker, 2005 ▶); data reduction: *SAINT*; program(s) used to solve structure: *SHELXS97* (Sheldrick, 2008 ▶); program(s) used to refine structure: *SHELXL97* (Sheldrick, 2008 ▶); molecular graphics: *SHELXTL* (Sheldrick, 2008 ▶); software used to prepare material for publication: *SHELXTL*.

## Supplementary Material

Crystal structure: contains datablock(s) I, global. DOI: 10.1107/S1600536812032266/su2479sup1.cif

Structure factors: contains datablock(s) I. DOI: 10.1107/S1600536812032266/su2479Isup2.hkl

Supplementary material file. DOI: 10.1107/S1600536812032266/su2479Isup3.cml

Additional supplementary materials:  crystallographic information; 3D view; checkCIF report

## Figures and Tables

**Table 1:** Hydrogen-bond geometry (Å, °)

*D*—H⋯*A*	*D*—H	H⋯*A*	*D*⋯*A*	*D*—H⋯*A*
C8—H8*B*⋯O1^i^	0.97	2.59	3.364 (3)	137
C10—H10*C*⋯O1^ii^	0.96	2.55	3.481 (4)	164

Symmetry codes: (i) 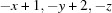; (ii) 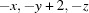.

**Fig. 1.**
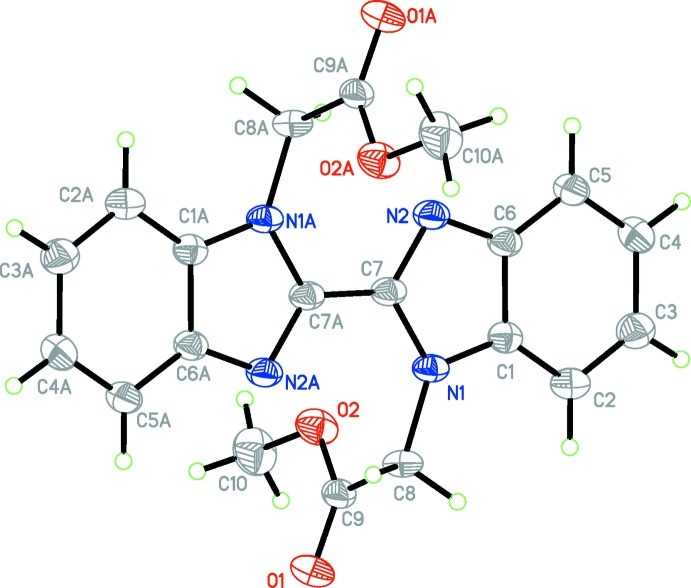
The molecular structure of the title molecule with the atom numbering [symmetry code for suffix A: -x, -y+1, -z+1]. Displacement ellipsoids are drawn at the 30% probability level

**Fig. 2.**
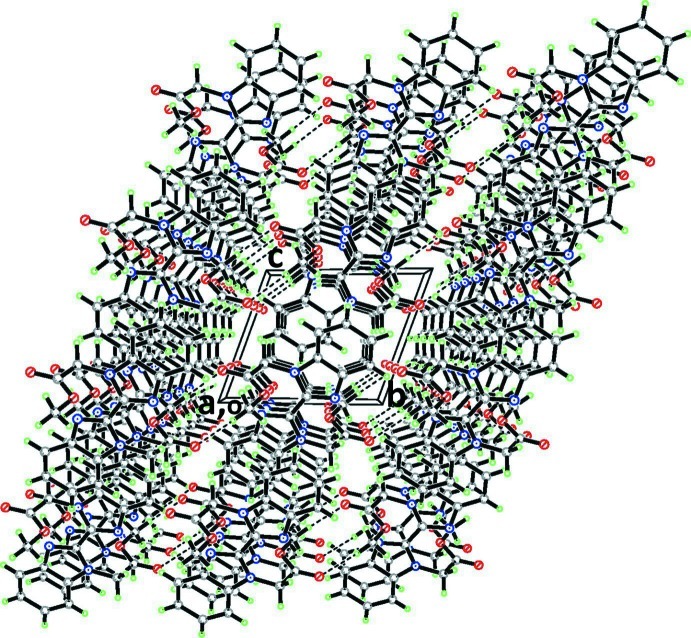
The crystal packing of the title compound, viewed along the b axis. Weak C—H···O interaction are shown as dashed lines.

## supplementary crystallographic information

### Comment

The whole molecule of the title compound (Fig.1) is generated by an inversion center. The benzimidazole system is essentially planar, with a dihedral angle of 0.8 (5)° between the planes of the benzene and imidazole rings. The benzimidazole ring make a dihedral angle of 89.4 (8)° with the plane passing through the acetate group (C9/O1/O2). This value is comparable to that observed in some similar structures (Al-Mohammed *et al.*, 2012; Fu *et al.*, 2009; Xu *et al.*, 2008).

In the crystal, weak intermolecular C—H···O hydrogen bonds (Table 1 and Fig. 2) and π–π stacking interactions [Cg1···Cg2^i^ = 3.743 (3) Å, where Cg1 is the centroid of ring N1/C1/C6/N2/C7; Cg2 is the centroid of ring C1-C6; symmetry code: (i) -x+1, -y+1, -z+1] stabilize the crystal structure.

### Experimental

The synthesis of 2,2'-bibenzimidazole [systematic name: 1*H*,1'*H*-2,2'-bibenzo[d]imidazole] has been reported (Tang *et al.*, 2007). A mixture of 2,2'-bibenzimidazole (11.71 g, 50 mmol) and NaOH (4.00 g, 100 mmol) in DMSO (40 mL) was stirred at 278 K for 2 h, and then methyl chloroacetate (10.85 g, 100 mmol) was added. The mixture was cooled to room temperature after stirring at 353 K for 24 h, and then poured into 200 mL of water. A yellow solid formed immediately, which was isolated by filtration. The crude product was then crystallized from methanol. Single crystals of the title compound, suitable for X-ray analysis, were obtained by slow evaporation of a solution in methanol.

### Refinement

The C-bound H-atoms were included in calculated positions and treated as riding atoms: C-H = 0.93, 0.96 and 0.97 Å for CH, CH_3_ and CH_2_ H-atoms, respectively, with U_iso_(H) = k × U_eq_(parent C-atom), where k = 1.5 for CH_3_ H-atoms and = 1.2 for other H-atoms.

### Crystal data

**Table:**

C_20_H_18_N_4_O_4_	*Z* = 1
*M**_r_* = 378.38	*F*(000) = 198
Triclinic, *P*1	*D*_x_ = 1.434 Mg m^−^^3^
Hall symbol: -P 1	Mo *K*α radiation, λ = 0.71073 Å
*a* = 6.904 (4) Å	Cell parameters from 853 reflections
*b* = 8.494 (5) Å	θ = 2.6–23.8°
*c* = 8.643 (5) Å	µ = 0.10 mm^−^^1^
α = 67.191 (5)°	*T* = 296 K
β = 70.360 (5)°	Block, brown
γ = 87.172 (5)°	0.33 × 0.31 × 0.29 mm
*V* = 438.1 (4) Å^3^	

### Data collection

**Table:**

Bruker APEXII CCD diffractometer	1600 independent reflections
Radiation source: fine-focus sealed tube	1172 reflections with *I* > 2σ(*I*)
Graphite monochromator	*R*_int_ = 0.027
φ and ω scans	θ_max_ = 25.5°, θ_min_ = 2.6°
Absorption correction: multi-scan (*SADABS*; Bruker, 2005)	*h* = −8→8
*T*_min_ = 0.967, *T*_max_ = 0.971	*k* = −9→10
3034 measured reflections	*l* = −10→10

### Refinement

**Table:**

Refinement on *F*^2^	Primary atom site location: structure-invariant direct methods
Least-squares matrix: full	Secondary atom site location: difference Fourier map
*R*[*F*^2^ > 2σ(*F*^2^)] = 0.048	Hydrogen site location: inferred from neighbouring sites
*wR*(*F*^2^) = 0.127	H-atom parameters constrained
*S* = 1.06	*w* = 1/[σ^2^(*F*_o_^2^) + (0.0656*P*)^2^ + 0.0023*P*] where *P* = (*F*_o_^2^ + 2*F*_c_^2^)/3
1600 reflections	(Δ/σ)_max_ < 0.001
128 parameters	Δρ_max_ = 0.14 e Å^−^^3^
0 restraints	Δρ_min_ = −0.25 e Å^−^^3^

### Special details

**Table:**

Geometry. All e.s.d.'s (except the e.s.d. in the dihedral angle between two l.s. planes) are estimated using the full covariance matrix. The cell e.s.d.'s are taken into account individually in the estimation of e.s.d.'s in distances, angles and torsion angles; correlations between e.s.d.'s in cell parameters are only used when they are defined by crystal symmetry. An approximate (isotropic) treatment of cell e.s.d.'s is used for estimating e.s.d.'s involving l.s. planes.
Refinement. Refinement of *F*^2^ against ALL reflections. The weighted *R*-factor *wR* and goodness of fit *S* are based on *F*^2^, conventional *R*-factors *R* are based on *F*, with *F* set to zero for negative *F*^2^. The threshold expression of *F*^2^ > σ(*F*^2^) is used only for calculating *R*-factors(gt) *etc*. and is not relevant to the choice of reflections for refinement. *R*-factors based on *F*^2^ are statistically about twice as large as those based on *F*, and *R*- factors based on ALL data will be even larger.

### Fractional atomic coordinates and isotropic or equivalent isotropic displacement parameters (Å^2^)

**Table:**

	*x*	*y*	*z*	*U*_iso_*/*U*_eq_	
C1	0.3492 (3)	0.6658 (2)	0.5117 (2)	0.0382 (5)	
C2	0.5275 (3)	0.7663 (3)	0.4646 (3)	0.0485 (6)	
H2	0.6033	0.8332	0.3460	0.058*	
C3	0.5865 (4)	0.7619 (3)	0.6022 (3)	0.0539 (6)	
H3	0.7063	0.8271	0.5762	0.065*	
C4	0.4722 (4)	0.6624 (3)	0.7808 (3)	0.0533 (6)	
H4	0.5173	0.6635	0.8704	0.064*	
C5	0.2955 (3)	0.5636 (3)	0.8265 (3)	0.0497 (6)	
H5	0.2197	0.4979	0.9454	0.060*	
C6	0.2327 (3)	0.5645 (2)	0.6892 (2)	0.0397 (5)	
C7	0.0742 (3)	0.5291 (2)	0.5281 (2)	0.0385 (5)	
C8	0.3089 (3)	0.7314 (2)	0.2179 (2)	0.0433 (5)	
H8A	0.2674	0.6583	0.1696	0.052*	
H8B	0.4585	0.7504	0.1691	0.052*	
C9	0.2220 (3)	0.9010 (3)	0.1547 (3)	0.0435 (5)	
C10	0.0081 (4)	1.1015 (3)	0.2334 (4)	0.0720 (8)	
H10A	0.1162	1.1933	0.1612	0.108*	
H10B	−0.0752	1.1195	0.3382	0.108*	
H10C	−0.0764	1.0989	0.1659	0.108*	
N1	0.2463 (2)	0.64117 (18)	0.41008 (19)	0.0388 (4)	
N2	0.0622 (3)	0.4797 (2)	0.6961 (2)	0.0435 (5)	
O1	0.2651 (3)	0.9897 (2)	−0.00051 (19)	0.0731 (6)	
O2	0.0980 (2)	0.94015 (17)	0.28611 (18)	0.0535 (5)	

### Atomic displacement parameters (Å^2^)

**Table:**

	*U*^11^	*U*^22^	*U*^33^	*U*^12^	*U*^13^	*U*^23^
C1	0.0432 (12)	0.0319 (10)	0.0357 (10)	0.0078 (9)	−0.0109 (9)	−0.0122 (8)
C2	0.0521 (14)	0.0445 (12)	0.0380 (11)	−0.0018 (10)	−0.0083 (10)	−0.0104 (9)
C3	0.0519 (14)	0.0543 (13)	0.0493 (13)	−0.0041 (11)	−0.0148 (11)	−0.0153 (11)
C4	0.0571 (15)	0.0605 (14)	0.0434 (12)	0.0081 (12)	−0.0224 (11)	−0.0176 (11)
C5	0.0498 (14)	0.0542 (13)	0.0354 (11)	0.0099 (11)	−0.0134 (10)	−0.0094 (9)
C6	0.0429 (13)	0.0351 (10)	0.0329 (10)	0.0077 (9)	−0.0102 (9)	−0.0079 (8)
C7	0.0420 (12)	0.0299 (10)	0.0321 (10)	0.0035 (9)	−0.0063 (8)	−0.0059 (8)
C8	0.0497 (13)	0.0421 (11)	0.0280 (10)	−0.0052 (10)	−0.0030 (9)	−0.0110 (8)
C9	0.0431 (13)	0.0446 (12)	0.0335 (10)	−0.0084 (9)	−0.0131 (9)	−0.0044 (9)
C10	0.082 (2)	0.0443 (13)	0.0862 (18)	0.0167 (13)	−0.0383 (16)	−0.0158 (13)
N1	0.0442 (10)	0.0304 (8)	0.0302 (8)	0.0015 (7)	−0.0059 (7)	−0.0058 (7)
N2	0.0454 (11)	0.0396 (9)	0.0321 (9)	0.0036 (8)	−0.0079 (8)	−0.0049 (7)
O1	0.0739 (12)	0.0779 (12)	0.0375 (9)	0.0000 (10)	−0.0172 (8)	0.0073 (8)
O2	0.0684 (11)	0.0384 (8)	0.0470 (9)	0.0120 (7)	−0.0185 (8)	−0.0119 (7)

### Geometric parameters (Å, º)

**Table:**

C1—N1	1.377 (3)	C7—N1	1.379 (2)
C1—C2	1.381 (3)	C7—C7^i^	1.449 (4)
C1—C6	1.398 (3)	C8—N1	1.448 (2)
C2—C3	1.368 (3)	C8—C9	1.503 (3)
C2—H2	0.9300	C8—H8A	0.9700
C3—C4	1.398 (3)	C8—H8B	0.9700
C3—H3	0.9300	C9—O1	1.194 (2)
C4—C5	1.367 (3)	C9—O2	1.321 (2)
C4—H4	0.9300	C10—O2	1.447 (3)
C5—C6	1.391 (3)	C10—H10A	0.9600
C5—H5	0.9300	C10—H10B	0.9600
C6—N2	1.383 (3)	C10—H10C	0.9600
C7—N2	1.320 (2)		
			
N1—C1—C2	131.59 (18)	N1—C8—C9	115.16 (16)
N1—C1—C6	105.60 (18)	N1—C8—H8A	108.5
C2—C1—C6	122.81 (19)	C9—C8—H8A	108.5
C3—C2—C1	116.29 (19)	N1—C8—H8B	108.5
C3—C2—H2	121.9	C9—C8—H8B	108.5
C1—C2—H2	121.9	H8A—C8—H8B	107.5
C2—C3—C4	122.0 (2)	O1—C9—O2	124.6 (2)
C2—C3—H3	119.0	O1—C9—C8	121.8 (2)
C4—C3—H3	119.0	O2—C9—C8	113.58 (15)
C5—C4—C3	121.4 (2)	O2—C10—H10A	109.5
C5—C4—H4	119.3	O2—C10—H10B	109.5
C3—C4—H4	119.3	H10A—C10—H10B	109.5
C4—C5—C6	117.83 (19)	O2—C10—H10C	109.5
C4—C5—H5	121.1	H10A—C10—H10C	109.5
C6—C5—H5	121.1	H10B—C10—H10C	109.5
N2—C6—C5	130.18 (18)	C1—N1—C7	106.58 (15)
N2—C6—C1	110.13 (17)	C1—N1—C8	123.60 (16)
C5—C6—C1	119.7 (2)	C7—N1—C8	129.65 (17)
N2—C7—N1	112.52 (18)	C7—N2—C6	105.16 (16)
N2—C7—C7^i^	124.2 (2)	C9—O2—C10	116.11 (17)
N1—C7—C7^i^	123.3 (2)		
			
N1—C1—C2—C3	179.33 (19)	C2—C1—N1—C8	−3.2 (3)
C6—C1—C2—C3	0.1 (3)	C6—C1—N1—C8	176.12 (16)
C1—C2—C3—C4	−0.5 (3)	N2—C7—N1—C1	−0.7 (2)
C2—C3—C4—C5	0.3 (3)	C7^i^—C7—N1—C1	−179.9 (2)
C3—C4—C5—C6	0.2 (3)	N2—C7—N1—C8	−176.09 (18)
C4—C5—C6—N2	−179.3 (2)	C7^i^—C7—N1—C8	4.8 (3)
C4—C5—C6—C1	−0.5 (3)	C9—C8—N1—C1	−87.6 (2)
N1—C1—C6—N2	0.0 (2)	C9—C8—N1—C7	87.1 (2)
C2—C1—C6—N2	179.38 (19)	N1—C7—N2—C6	0.7 (2)
N1—C1—C6—C5	−179.02 (17)	C7^i^—C7—N2—C6	179.9 (2)
C2—C1—C6—C5	0.4 (3)	C5—C6—N2—C7	178.5 (2)
N1—C8—C9—O1	178.39 (19)	C1—C6—N2—C7	−0.4 (2)
N1—C8—C9—O2	−1.1 (3)	O1—C9—O2—C10	1.1 (3)
C2—C1—N1—C7	−178.9 (2)	C8—C9—O2—C10	−179.49 (17)
C6—C1—N1—C7	0.42 (19)		

Symmetry code: (i) −*x*, −*y*+1, −*z*+1.

### Hydrogen-bond geometry (Å, º)

**Table:**

*D*—H···*A*	*D*—H	H···*A*	*D*···*A*	*D*—H···*A*
C8—H8*B*···O1^ii^	0.97	2.59	3.364 (3)	137
C10—H10*C*···O1^iii^	0.96	2.55	3.481 (4)	164

Symmetry codes: (ii) −*x*+1, −*y*+2, −*z*; (iii) −*x*, −*y*+2, −*z*.
